# Supplementary material for: One million years of glaciation and denudation history in west Greenland
Source: Nat Commun. 2017 Jan 18;8:14199. doi: 10.1038/ncomms14199 (PMC5253681; doi:10.1038/ncomms14199)
Supplement: Supplementary Information — Supplementary Figures and Supplementary References [file ncomms14199-s1.pdf]

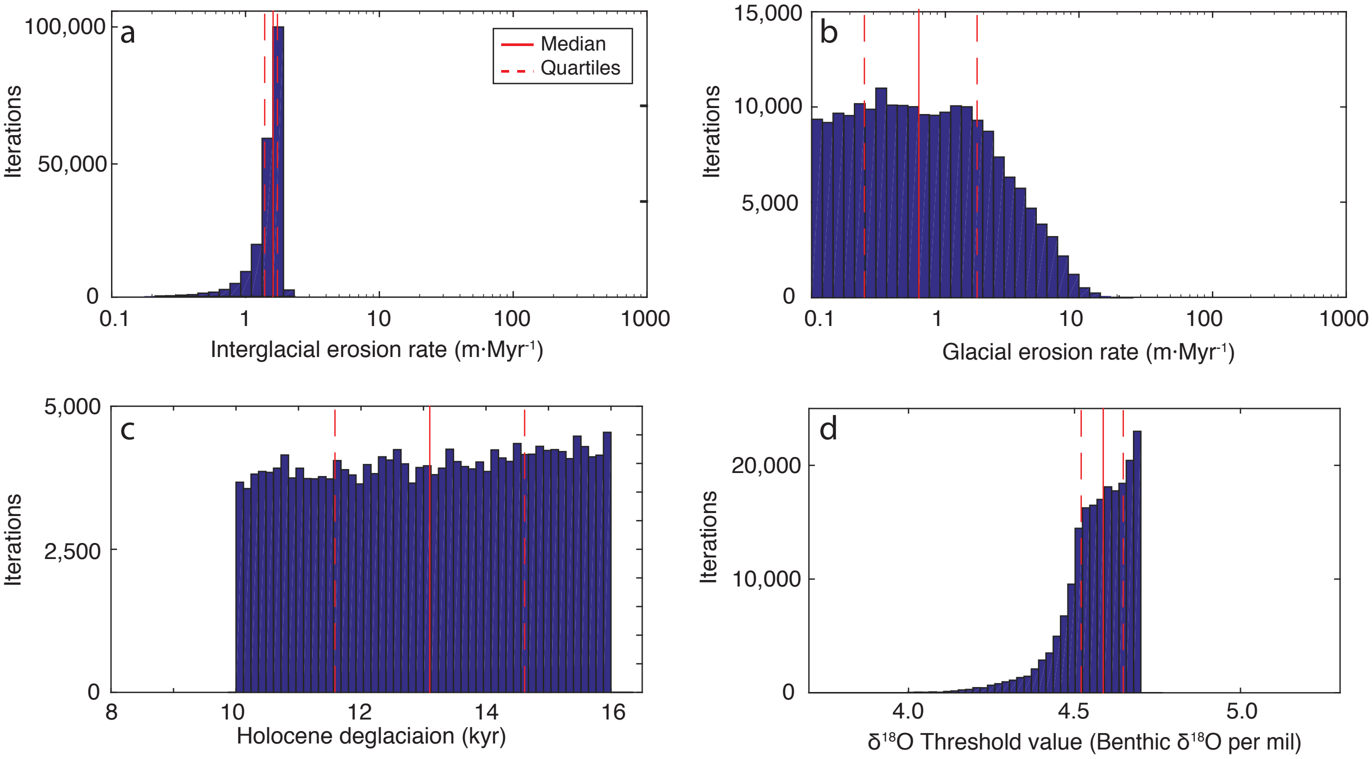

Supplementary Figure 1: Distribution of parameter values for one sample. The four free parameters used in the MCMC inversion to find the most likely exposure- and denudation history. For each sample we initiate 200,000 iterations. This figure presents are results from sample 13-GROR-70 from Uummannaq[1]. A red solid line marks the median values and red dashed lines mark the first and third quartiles. (**a**) and (**b**) distributions of possible interglacial and glacial erosion rates for the measured TCN concentration. (**c**) shows the timing of the Holocene deglaciation, which in this case is limited by the interval 10-16 kyr BP. (**d**) distribution of possible δ^{18}O-threshold values that yield exposure histories in agreement with the measured TCN concentrations.

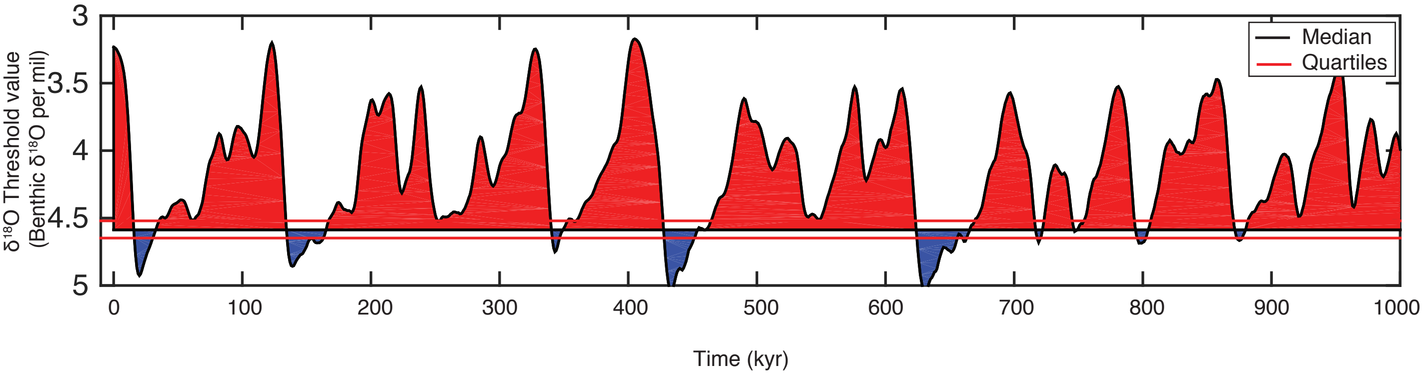

Supplementary Figure 2: Exposure history of sample 13-GROR-70 from Uummannaq[1] determined by applying a threshold value to the global marine benthic $\delta^{18}O$ stack[2]. The $\delta^{18}O$ value is a proxy for global ice volume and the applied threshold (horizontal black line) defines the periods of full exposure, i.e. no ice cover (red), and periods of complete burial, i.e. thick ice cover (blue). Red lines mark the first and third quartiles of the estimated threshold value.

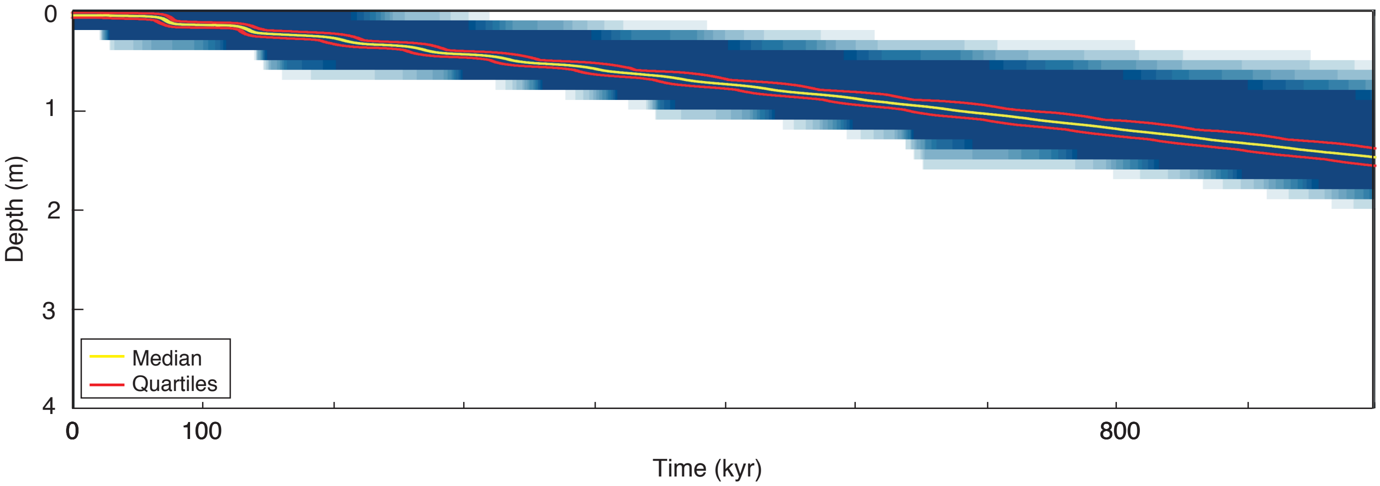

Supplementary Figure 3: Exhumation history. Possible exhumation histories based on 200,000 iterations of sample 13-GROR-70 from Uummannaq[1] (blue lines), which all give a TCN end concentration matching the measured values. Red lines mark the 25% and 75% quartiles, the yellow line marks the median value. Note that the total denudation rate is very well constrained for the large majority of samples, even though the individual interglacial and glacial erosion rates in some cases are relatively poorly constrained. This basically reflects the fact that two TCNs (^{10}Be and ^{26}Al) are not always sufficient to perfectly constrain both erosion rates because different landscape histories may result in the same TCN concentrations. The integrated erosion over longer time periods, such as 1 Myr is, however, well constrained. The overall uncertainty on the estimated denudation rate generally increases with denudation rate.

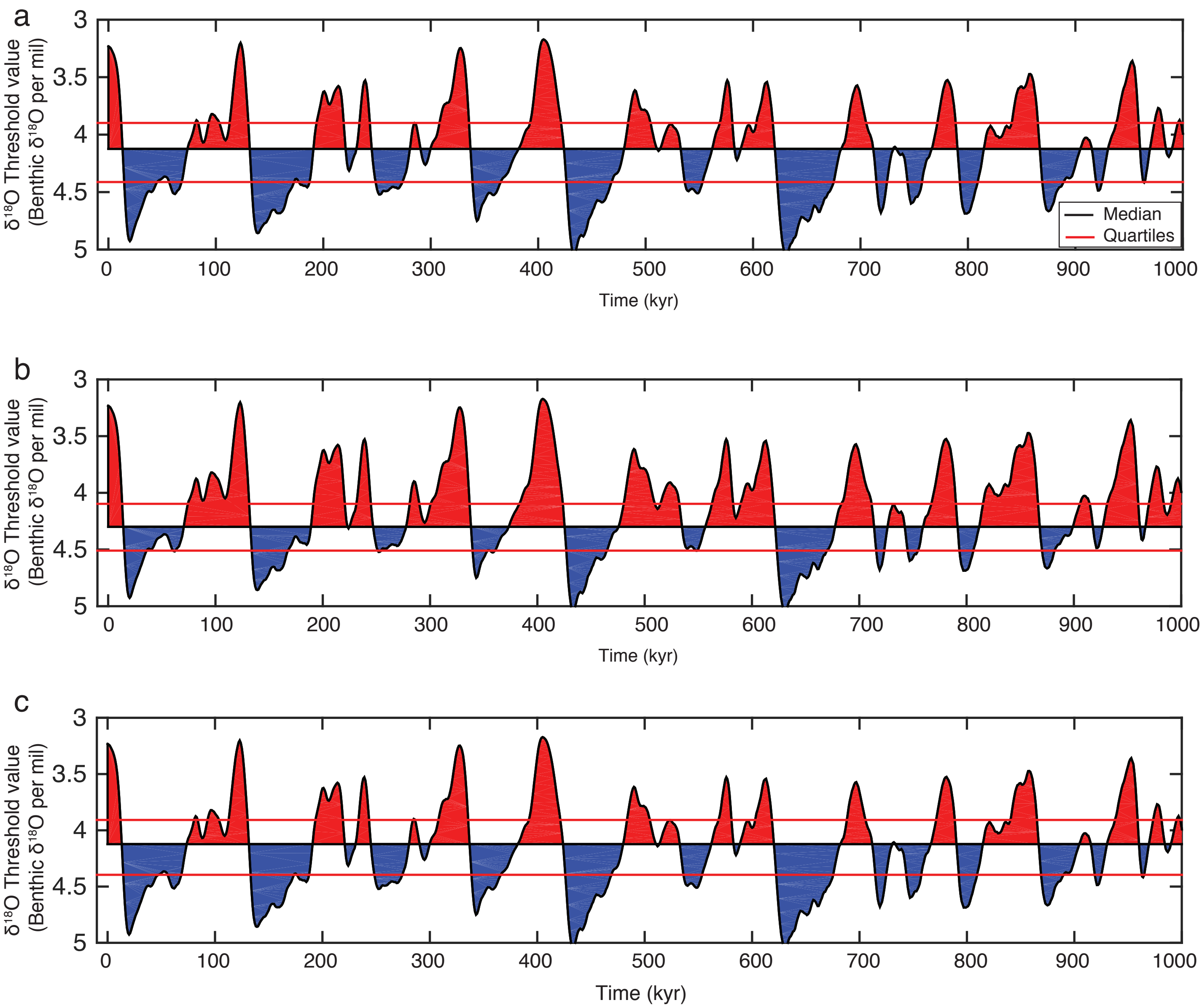

Supplementary Figure 4: Compiled exposure histories for the three areas in West Greenland based on the average exposure histories of the individual bedrock samples at each site. (**a**) exposure history for Upernavik based on 11 samples[3]. (**b**) exposure history for Uummannaq based on 12 samples[1,4]. (**c**) exposure history for Sisimiut (Sukkertoppen and Itilleq) based on 16 samples[1,5,6]. The uncertainties are represented as the mean of the 25th and 75th quartiles of all the samples at each site. It would be possible to significantly increase the precision of the threshold value by including other TCNs, such as ^{14}C and ^{21}Ne, and/or by collecting samples from a depth profile, but no such data is currently available.

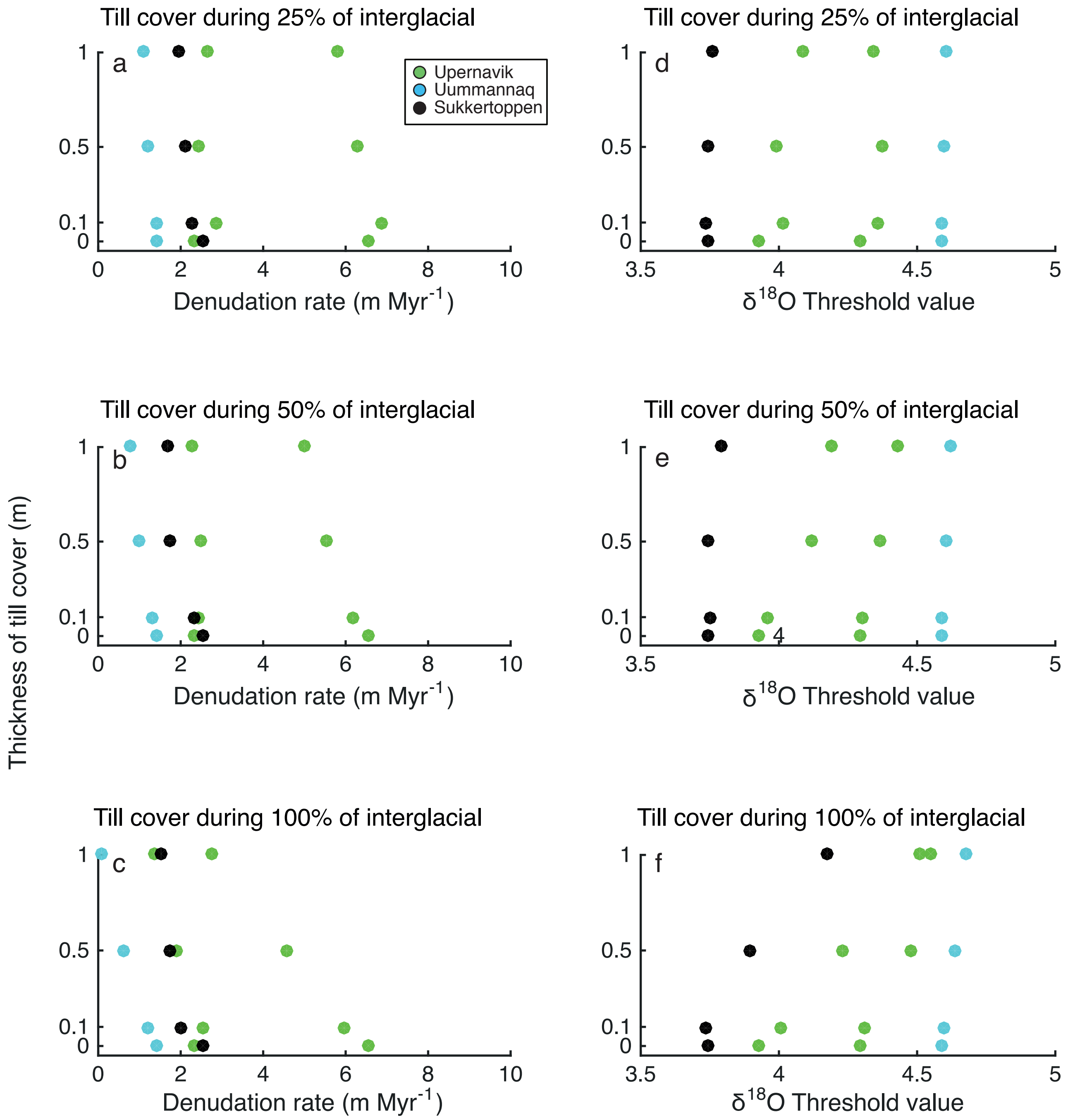

Supplementary Figure 5: Effect of till cover on model results based on four samples. (**a-c**) Denudation rate under till thicknesses varying from 0.1 m to 1.0 m, covering the bedrock during 25%, 50%, and 100% of the interglacial period. The lowermost data point represents the sample results without any till cover. (**d-f**) $\delta^{18}O$ threshold value under till thick-nesses varying from 0.1 m to 1.0 m, covering the bedrock during 25%, 50%, and 100% of the interglacial period. The lowermost data point represents the sample results without any till cover. Error bars are defined as the first and third quartiles of the 200,000 iterations per sample.

Supplementary References

1. Beel, C. R., Lifton, N. A., Briner, J. P. & Goehring, B. M. Quaternary evolution and ice sheet history of contrasting landscapes in Uummannaq and Sukkertoppen, western Greenland. *Quat. Sci. Rev.* **149,** 248–258 (2016).
2. Lisiecki, L. E. & Raymo, M. E. A Pliocene-Pleistocene stack of 57 globally distributed benthic $\delta^{18}O$ records. *Paleoceanography* **20,** 1–17 (2005).
3. Corbett, L. B., Bierman, P. R., Graly, J. A., Neumann, T. A. & Rood, D. H. Constraining landscape history and glacial erosivity using paired cosmogenic nuclides in Upernavik, Northwest Greenland. *Bull. Geol. Soc. Am.* **125,** 1539–1553 (2013).
4. Roberts, D. H. *et al.* Ice sheet extent and early deglacial history of the southwestern sector of the Greenland Ice Sheet. *Quat. Sci. Rev.* **28,** 2760–2773 (2009).
5. Roberts, D. H., Rea, B. R., Lane, T. P., Schnabel, C. & Rodés, A. New constraints on Greenland ice sheet dynamics during the last glacial cycle: Evidence from the Uummannaq ice stream system. *J. Geophys. Res. Earth Surf.* **118,** 519–541 (2013).
6. Lane, T. P., Roberts, D. H., Rea, B. R., Cofaigh, C. & Vieli, A. Controls on bedrock bedform development beneath the Uummannaq Ice Stream onset zone, West Greenland. *Geomorphology* **231,** 301–313 (2015).
